# Ulnar-evoked motor activity recorded over the abductor pollicis brevis region in carpal tunnel syndrome: a preliminary case series

**DOI:** 10.3389/fnhum.2026.1833401

**Published:** 2026-09-03

**Authors:** Beatrix Vághy, Szabolcs Várbíró

**Affiliations:** 1 Doctoral School, Semmelweis University, Budapest, Hungary; 2 Department of Obstetrics and Gynecology, University of Szeged (SZTE), Szeged, Hungary

**Keywords:** abductor pollicis brevis, carpal tunnel syndrome, median nerve, median–ulnar communication, nerve conduction studies, Riche–Cannieu anastomosis, thenar muscles, ulnar nerve

## Abstract

**Objective:**

To describe a brief and easily applicable electroneurographic (ENG) maneuver that can reveal ulnar-evoked motor activity detectable over the abducto’r pollicis brevis (APB) region in selected carpal tunnel syndrome (CTS) cases. The aim is to illustrate how targeted ulnar stimulation may complement routine CTS evaluation, rather than to redefine severity grading or clinical decision making.

**Methods:**

Routine upper-limb nerve conduction studies were supplemented with targeted motor stimulation of the ulnar nerve at the wrist, elbow, and above the elbow while recording over the APB region. From a larger CTS cohort, three illustrative cases with distinct electrophysiological patterns were retrospectively selected to demonstrate ulnar-evoked activity detectable at the APB recording site across selected CTS severity categories.

**Results:**

In all three cases, ulnar nerve stimulation elicited reproducible compound muscle action potentials recorded over the APB region, including situations where median-evoked APB responses were markedly reduced or absent. Latency characteristics and proximodistal consistency were compatible with known patterns of median–ulnar motor communication and demonstrated how ulnar-evoked activity detectable at the APB site may appear across the chosen CTS severity categories.

**Conclusion:**

This preliminary case series shows that targeted ulnar stimulation can reveal ulnar-evoked motor activity detectable over the APB recording site in selected CTS patients. These descriptive findings raise the hypothesis that median motor parameters alone may not fully reflect the pattern of motor activity measurable at the APB region in all individuals.

**Significance:**

The maneuver is simple and quick, and may provide supplementary neurophysiological information in CTS—particularly in cases with reduced or absent median-evoked APB responses. Larger studies are required to determine its reproducibility, prevalence, and clinical relevance.

## 1 Introduction

Carpal tunnel syndrome (CTS) is the most common entrapment neuropathy of the upper limb and is routinely evaluated using nerve conduction studies. Electrophysiological assessment typically relies on median sensory and motor conduction parameters, particularly responses recorded from the abductor pollicis brevis (APB), which is traditionally regarded as a median nerve–innervated muscle (Werner and Andary, 2011; Padua et al., 2016).

Interpretation of APB recordings may occasionally be complicated by anatomical variations in the motor innervation of the thenar region. Median–ulnar neural communications are common in the upper limb, and several studies have demonstrated that motor fibers originating from the ulnar nerve may contribute to thenar muscle activation through intrapalmar neural connections, most notably the Riche–Cannieu anastomosis (Roy et al., 2016; Caetano et al., 2018).

Recognition of such variants is particularly important when evaluating CTS patients with markedly reduced or absent median-evoked APB responses. In these situations, electrophysiological activity recorded over the thenar region may not necessarily reflect median motor function alone. Previous reports have described electrophysiological findings compatible with ulnar contributions to thenar activation and have highlighted the potential diagnostic implications of median–ulnar motor communications (Wali et al., 2017; Di Stefano et al., 2021; Alajmi et al., 2022).

The present preliminary case series describes a simple electrophysiological maneuver in which the ulnar nerve is stimulated while recording over the APB region. Three illustrative CTS cases were selected to demonstrate reproducible ulnar-evoked motor activity measurable at the APB recording site across different CTS severity grades. The aim of this report is not to redefine CTS severity assessment or establish diagnostic criteria, but to illustrate a potentially useful supplementary observation that may assist interpretation of routine nerve conduction studies. Median–ulnar motor communications and their anatomical variants have been extensively described in classical neurophysiology and anatomical literature (Brown and Landau, 2013; Kimura, 1983; Riveros et al., 2019; Sarikcioglu and Sindel, 2002; Yang et al., 2016). These variants may influence the interpretation of motor conduction studies and should be considered when evaluating atypical ENG/NCS patterns.

## 2 Anatomical background

The motor innervation of the thenar musculature exhibits substantial anatomical variability. Although the APB is classically innervated by the recurrent motor branch of the median nerve, anatomical studies have demonstrated frequent connections between the median and ulnar nerves within the hand (Harness and Sekeles, 1971; Caetano et al., 2018).

The best-known intrapalmar communication is the Riche–Cannieu anastomosis, which connects the deep branch of the ulnar nerve with branches of the median nerve supplying the thenar muscles. A meta-analysis by Roy et al. reported a pooled prevalence of approximately 55% for Riche–Cannieu communications, although reported frequencies vary across anatomical and electrophysiological studies (Roy et al., 2016).

Because of these interconnections, motor activity recorded over the thenar region may, in some individuals, reflect contributions from both median- and ulnar-derived motor pathways. Such anatomical variability provides a plausible substrate for ulnar-evoked motor activity detectable at the APB recording site and underscores the importance of considering alternative motor inputs when interpreting electrophysiological findings in CTS.

## 3 Methods

### 3.1 Study design and setting

This preliminary retrospective case series was derived from routine upper-limb electroneurography (ENG) examinations performed between January 2023 and March 2025 in two accredited neurophysiology laboratories. The objective was to identify carpal tunnel syndrome (CTS) cases in which targeted ulnar nerve stimulation elicited reproducible motor activity detectable over the abductor pollicis brevis (APB) recording region.

### 3.2 Study population and case selection

CTS severity was classified according to standard electrodiagnostic criteria based on sensory and motor conduction abnormalities and categorized as mild, moderate, or severe. Mild CTS was defined by mildly prolonged median distal motor latency with preserved CMAP amplitude, together with reduced distal sensory conduction velocity and preserved or mildly reduced sensory amplitude. Moderate CTS was defined by prolonged median distal motor latency, reduced CMAP amplitude, and reduced distal sensory conduction velocity and sensory amplitude. Severe CTS was defined by markedly prolonged median distal motor latency, substantially reduced or absent CMAP amplitude, and severely reduced or absent sensory amplitude with markedly reduced distal sensory conduction velocity.

During the study period, 89 consecutive upper-limb ENG examinations were performed for suspected CTS. Of the examined limbs, 61 fulfilled established electrophysiological criteria for CTS (Werner and Andary, 2011). Among the 61 CTS-positive limbs, reproducible ulnar-evoked motor activity recorded over the APB region was identified in 28 limbs (45.9%).

From this subgroup, three cases were retrospectively selected for detailed presentation. The selected cases represented distinct electrophysiological patterns:

1. Mild CTS with a small ulnar-evoked APB-region response;
2. Moderate CTS with prominent ulnar-evoked APB-region activity;
3. Severe CTS with strong ulnar-evoked APB-region activity.

These cases were chosen to illustrate electrophysiological variability rather than to estimate prevalence, diagnostic accuracy, or associations between CTS severity and the magnitude of ulnar-evoked responses.

Skin temperature and stimulation parameters: Skin temperature was continuously monitored using a dedicated surface temperature electrode integrated into the ENG system and was maintained between 34 °C and 36 °C throughout the examination. The 0.0 °C value displayed in the waveform outputs represents an unfilled software field rather than the actual measured skin temperature. The distal wrist stimulation distance for median motor studies was consistently 8 cm, as documented in the waveform records.

### 3.3 Physiological comparator dataset

An internal physiological comparator dataset was derived from the CTS-negative subset of the same consecutive ENG series. Twenty-eight limbs showed no electrophysiological evidence of CTS, ulnar neuropathy, polyneuropathy, or other peripheral nerve disorders. The comparator cohort comprised 15 right-sided and 13 left-sided limbs. Mean age was 47.8 years (range 25–69 years), and 23 subjects were female and 5 male. Using the same stimulation and recording configuration as in the illustrative CTS cases, ulnar stimulation with recording over the APB region was performed in all comparator limbs to characterize the electrophysiological properties of motor activity detectable at this recording site. Of the 28 CTS-negative limbs, 13 demonstrated reproducible ulnar-evoked APB-region responses suitable for quantitative latency and amplitude analysis. In the remaining limbs, the recorded potentials did not meet the reproducibility or morphological criteria required for evaluation.

All latency and amplitude calculations reported for the physiological comparator dataset were based on these 13 limbs demonstrating reproducible APB-region responses.

This CTS-negative cohort served exclusively as a physiological comparator for interpretation of the illustrative CTS cases and was not intended to estimate the prevalence of ulnar-derived APB-region responses in the general population. The proportion observed in this study (13/28; 46.4%) is consistent with international anatomical and electrophysiological literature, which reports median–ulnar intrapalmar motor communications — including the Riche–Cannieu anastomosis — in approximately 50–60% of individuals (Roy et al., 2016; Caetano et al., 2018). Because this cohort was retrospective and not designed as a normative reference population, the reported latency and amplitude values were used descriptively to provide physiological context rather than formal diagnostic limits.

### 3.4 Electrophysiological procedures

Routine ENG included median sensory conduction recorded antidromically from digit II, median motor conduction recorded over the APB with stimulation at the wrist and antecubital fossa, and ulnar motor conduction recorded over the abductor digiti minimi (ADM) with stimulation at the wrist, below the elbow, and above the elbow.

Median nerve stimulation sites were located 3 cm proximal to the pisiform at the wrist and in the antecubital fossa lateral to the biceps tendon. Ulnar nerve stimulation sites were located 3 cm proximal to the pisiform at the wrist, 2 cm distal to the medial epicondyle, and 10 cm proximal to the medial epicondyle.

Skin temperature was continuously monitored using a dedicated surface temperature electrode integrated into the ENG system and was maintained between 34 °C and 36 °C throughout the examination. Filter settings were 2 Hz–10 kHz. Stimuli consisted of 0.2 ms square-wave pulses delivered at supramaximal intensity throughout the examination.

### 3.5 Targeted ulnar stimulation protocol

To assess potential ulnar-derived motor activity detectable over the APB recording region, routine ENG was supplemented with an additional stimulation protocol.

The ulnar nerve was stimulated at the wrist, below the elbow, and above the elbow using the same stimulation sites employed during routine ulnar motor conduction studies. Surface recording electrodes were placed over the APB region in a standard belly-tendon montage, with the reference electrode positioned on the proximal phalanx of the thumb and the ground electrode on the dorsum of the hand.

Proximodistal stimulation was used to assess latency progression and to reduce potential misinterpretation related to proximal median-ulnar crossover variants such as Martin-Gruber or Marinacci communications.

### 3.6 Interpretation of APB-region responses

Ulnar-evoked responses recorded over the APB region were interpreted using the internal physiological comparator dataset.

Responses were considered compatible with ulnar-derived motor activity when they demonstrated:

1. Reproducible CMAP morphology;
2. Physiological latency characteristics comparable to those observed in the comparator cohort;
3. Persistence during proximal stimulation;
4. Appropriate proximodistal latency progression.

These features support the presence of a localized physiological motor source detectable from the APB recording position. However, surface recordings do not permit definitive identification of the activated muscle. Accordingly, the recorded responses were interpreted as ulnar-evoked motor activity detectable over the APB region rather than proof of APB-specific activation. Contributions from adjacent ulnar-innervated muscles, including the adductor pollicis, deep head of the flexor pollicis brevis, first dorsal interosseous, or other intrinsic hand muscles, cannot be excluded.

### 3.7 Statistical analysis

Given the descriptive nature of this preliminary case series, no formal hypothesis testing was performed. Data from the physiological comparator cohort are presented as mean ± standard deviation and range and were used solely to provide physiological context for interpretation of the illustrated cases.

### 3.8 Ethical considerations

All procedures adhered to the Declaration of Helsinki and were approved by the Hungarian Medical Research Council (ETT TUKEB; approval number 848/2022). All participants provided written informed consent for electrophysiological examination and scientific use of anonymized clinical data.

All examinations were performed in two accredited neurophysiology laboratories by board-certified clinical neurophysiologists trained in electrodiagnostic medicine.

The original ENG recordings and clinical documentation were independently reviewed by two experienced examiners. Because of the retrospective study design, blinded assessment was not feasible. All data were anonymized prior to analysis, and access was restricted to authorized personnel in accordance with institutional and national data protection regulations.

Patients were referred by neurologists, rheumatologists, or hand surgeons with suspected CTS. To minimize confounding factors affecting peripheral nerve conduction, individuals with diabetes mellitus, rheumatoid arthritis, peripheral polyneuropathy, prior hand surgery, or markedly low pain tolerance were excluded.

## 4 Results

A total of 61 electrophysiologically confirmed CTS cases were identified during the study period. From this cohort, three representative cases were selected to illustrate distinct patterns of median motor involvement and ulnar-evoked activity detectable over the abductor pollicis brevis (APB) region. These cases demonstrate the electrophysiological spectrum from preserved median motor responses with small ulnar-evoked APB-region activity (mild CTS), through reduced median activation accompanied by prominent ulnar-evoked activity (moderate CTS), to markedly impaired median motor input with strong ulnar-evoked activity at the APB recording site (severe CTS).

### 4.1 Reference ulnar motor latency

Physiological ulnar-evoked responses recorded over the APB region were characterized using the internal comparator dataset derived from CTS-negative limbs. These recordings demonstrated characteristic distal motor latency values, well-formed CMAP amplitudes, sharply contoured M-waves, and reproducibility with proximal stimulation.

Distal motor latency showed a mean of 3.1 ms (SD 0.4 ms), a median of 3.0 ms, and a range of 2.4–4.0 ms. CMAP amplitudes demonstrated a mean of 3.39 mV (SD 1.45 mV), a median of 3.30 mV, and a range of 0.33–7.90 mV.

These measurements served as a physiological comparator for interpretation of ulnar-evoked responses observed in the illustrative CTS cases. Because recordings obtained over the APB region may include contributions from adjacent ulnar-innervated muscles, the comparator dataset was used to characterize the electrophysiological properties of responses recorded at this electrode position rather than to establish APB-specific innervation.

As the comparator cohort was retrospective and not designed as a normative reference population, the observed latency and amplitude values were used descriptively rather than as formal normal limits or diagnostic thresholds.

#### 4.1.1 Case 1—mild CTS with a small ulnar-evoked APB-region response

A 46-year-old woman presented with intermittent paresthesias in the median nerve distribution and preserved thumb abduction strength. Electrophysiological examination was consistent with mild carpal tunnel syndrome, characterized by sensory conduction slowing with preserved sensory and motor response amplitudes. Routine ulnar sensory and motor conduction studies were within normal limits.

Targeted stimulation of the ulnar nerve while recording over the APB region elicited a small but reproducible compound muscle action potential. The response demonstrated stable morphology, remained detectable during proximal stimulation, and showed proximodistal consistency. Although the latency characteristics differed slightly from those observed in the internal physiological comparator dataset, the waveform was reproducible and sharply contoured. These findings support the presence of ulnar-evoked motor activity detectable at the APB recording site despite largely preserved median motor function.

**Figure 1:**
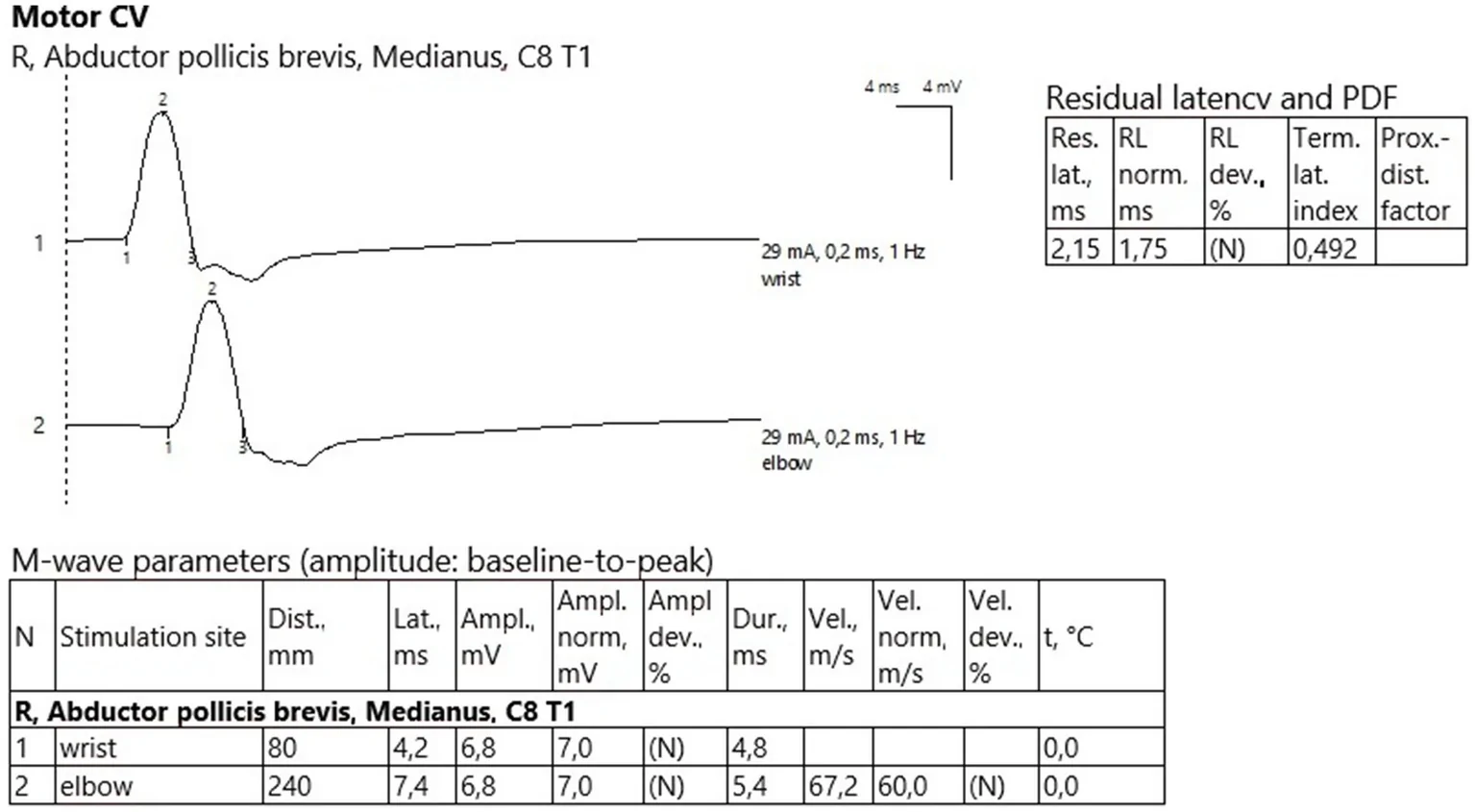
Medianus → APB motor response (Case 1, mild CTS). Compound muscle action potentials (CMAPs) recorded over the Abductor Pollicis Brevis (APB) following Median nerve stimulation at the wrist and elbow. Distal motor latency was prolonged (4.2 ms) with preserved amplitude (6.8 mV), consistent with mild CTS without motor axon loss. Waveforms demonstrated stable morphology and reproducibility across stimulation sites.

**Figure 2:**
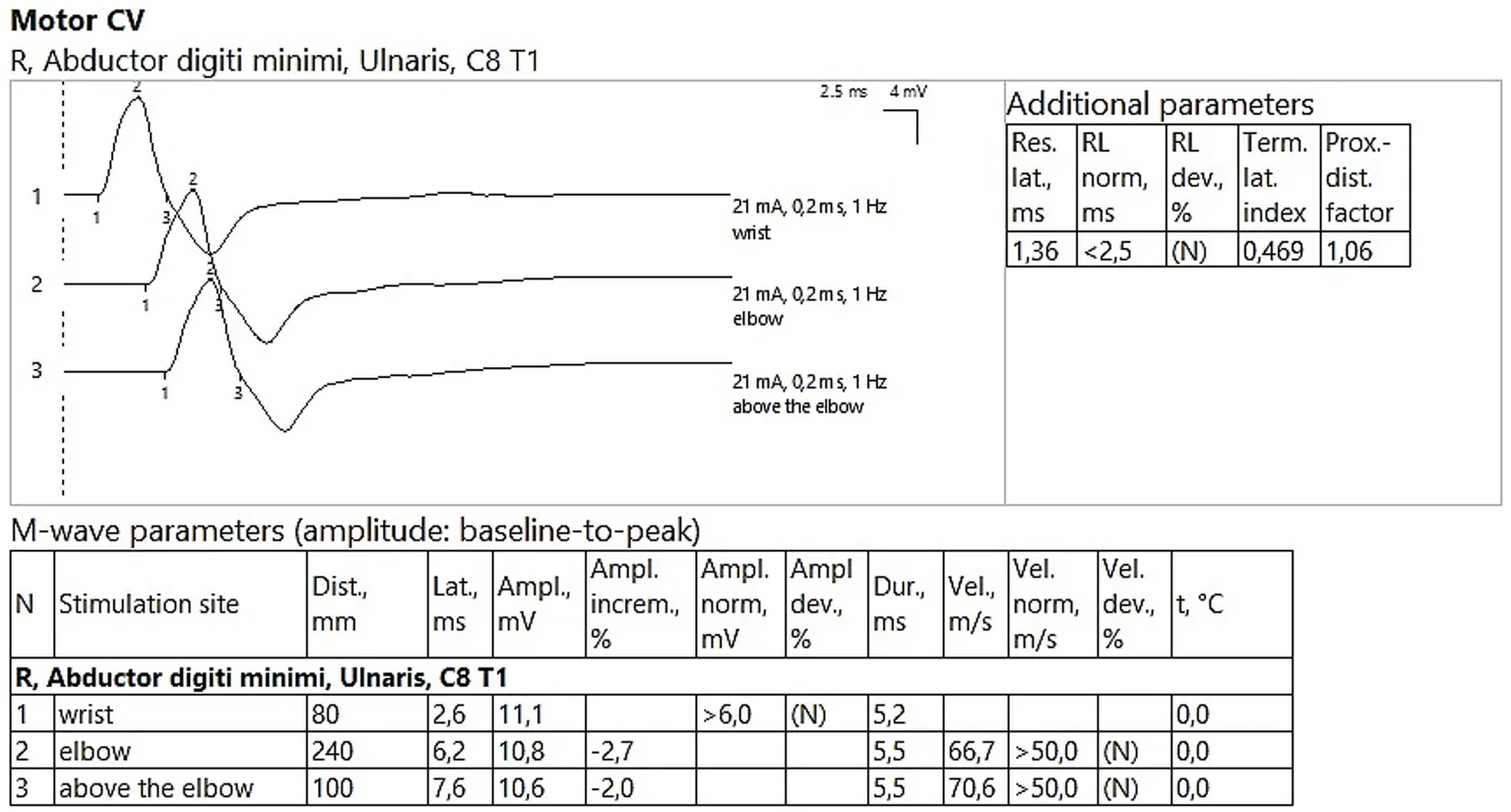
Ulnaris → ADM motor conduction (Case 1, mild CTS). Ulnar CMAPs recorded over the abductor digiti minimi (ADM) with stimulation at the wrist, 2 cm distal to the medial epicondyle, and 10 cm proximal to the medial epicondyle. Distal latency (2.6 ms), amplitude (11.1 mV), and conduction velocities (66.7–70.6 m/s) were within normal limits, confirming preserved ulnar motor function.

**Figure 3:**
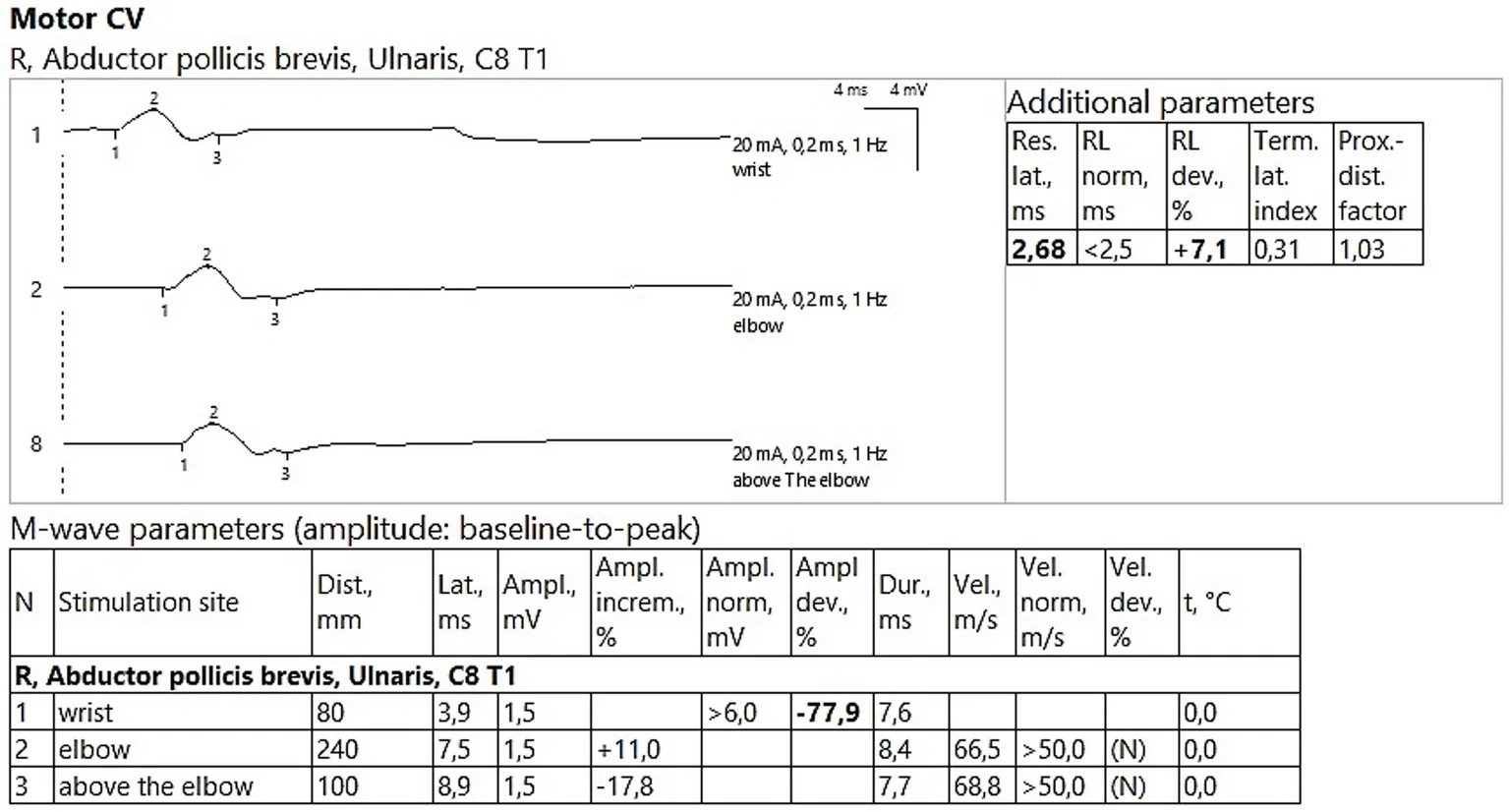
Ulnaris → APB region motor response (Case 1) Ulnar nerve stimulation at the wrist, 2 cm distal to the medial epicondyle, and 10 cm proximal to the medial epicondyle elicited a small but reproducible CMAP recorded over the APB region. Distal motor latency was 3.9 ms, amplitude 1.5 mV, and wrist–elbow conduction velocity 66.5 m/s, indicating a physiologically meaningful ulnar-evoked motor signal detectable at the APB recording site.

Waveform documentation (Figures 1–3). Medianus → APB (Figure 1): prolonged distal motor latency with preserved amplitude. Ulnaris → ADM (Figure 2): normal ulnar motor conduction parameters. Ulnaris → APB (Figure 3): small but reproducible ulnar-evoked APB-region activity (Table 1).

**Table 1:** Clinical and electrophysiological characteristics of the three illustrative CTS cases with ulnar-evoked activity recorded over the APB region.

Parameter	Case 1 (mild CTS)	Case 2 (moderate CTS)	Case 3 (severe CTS)
Age (years)	46	52	61
Sex	Female	Female	Male
Side	Right	Right	Left
Median sensory CV (m/s)	48.6	32.4	Not obtainable
Median sensory SNAP amplitude (μV)	7.1	2.5	Absent
Median motor APB DML (ms)	4.2	5.4	7.2
Median motor APB CMAP amplitude (mV)	6.8	5.7	2.2
Median motor APB CV (m/s)	67.2	55.3	52.9
Ulnar sensory CV (m/s)	58.0	58.8	70.2
Ulnar sensory SNAP amplitude (μV)	17.1	13.1	25.8
Ulnar motor ADM DML (ms)	2.6	2.4	2.6
Ulnar motor ADM CMAP amplitude (mV)	11.1	11.5	10.8
Ulnar motor ADM CV (m/s)	66.7	73.6	68.2
Ulnar → APB DML (ms)	3.9	3.8	3.4
Ulnar → APB wrist amplitude (mV)	1.5	3.2	4.0
Ulnar → APB elbow amplitude (mV)	1.5	3.2	4.1
Ulnar → APB above-elbow amplitude (mV)	1.5	3.1	4.1
Ulnar → APB CV wrist-elbow (m/s)	66.5	73.7	72.3
Ulnar → APB CV elbow-above elbow (m/s)	68.8	71.5	71.4
CTS severity	Mild	Moderate	Severe

#### 4.1.2 Case 2—moderate CTS with prominent ulnar-evoked APB-region activity

A 52-year-old woman presented with persistent median nerve symptoms and mildly reduced thumb abduction strength. Electrophysiological examination demonstrated moderate carpal tunnel syndrome with both sensory and motor median nerve involvement. Median sensory abnormalities were more pronounced than in Case 1, while routine ulnar sensory and motor conduction studies remained normal.

Targeted ulnar stimulation produced a stable, sharply contoured, and reproducible response recorded over the APB region. The latency characteristics were comparable to those observed in the internal physiological comparator dataset, and the response remained readily detectable with proximal stimulation. Compared with Case 1, the ulnar-evoked APB-region response was more prominent, despite only moderate median motor impairment. These findings illustrate that substantial ulnar-evoked activity detectable over the APB region may coexist with moderate CTS.

**Figure 4:**
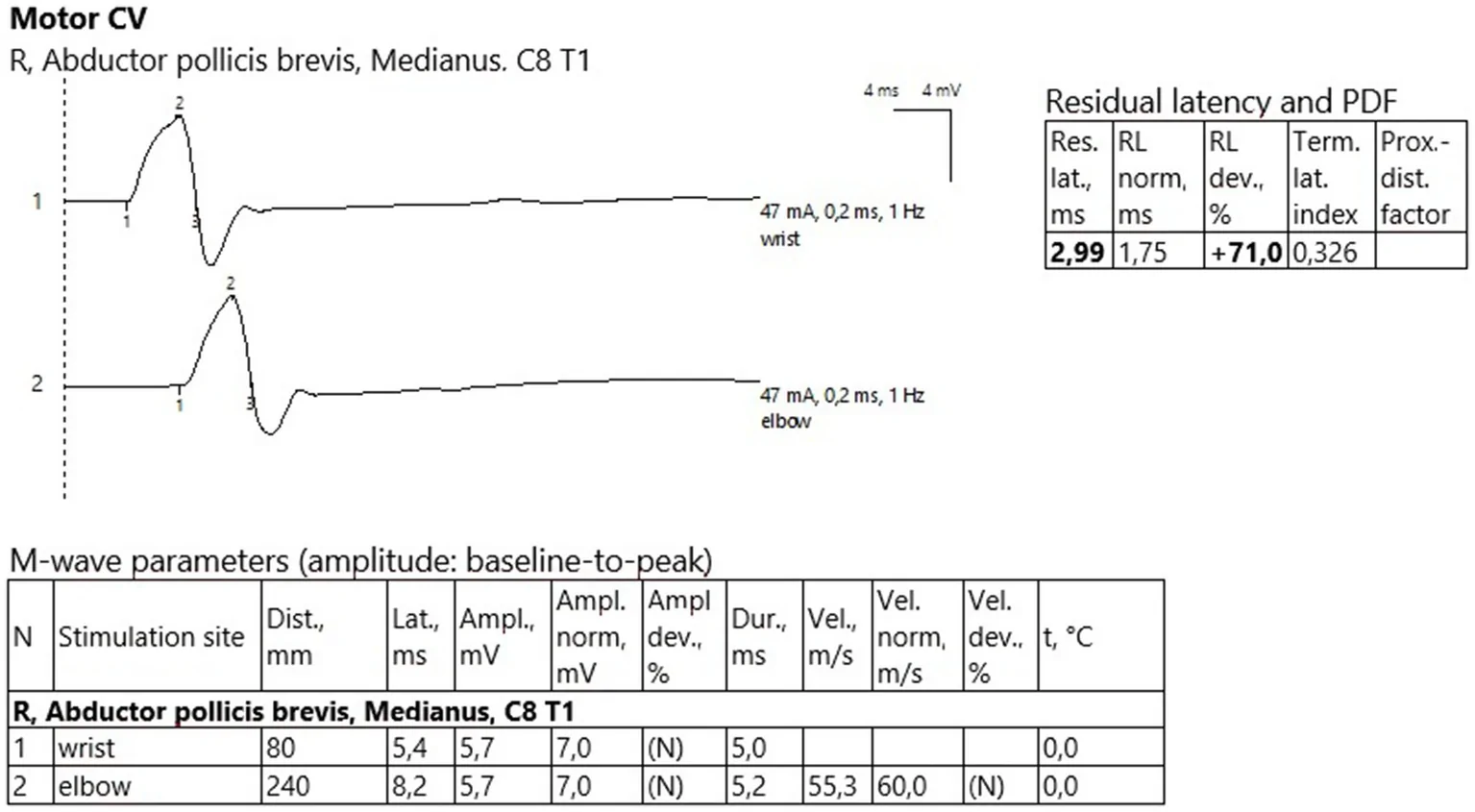
Medianus → APB motor response (Case 2) Median nerve stimulation at the wrist and elbow elicited a CMAP recorded over the APB. Distal motor latency was 5.4 ms, amplitude 5.7 mV, and wrist–elbow conduction velocity 55.3 m/s, consistent with moderate CTS.

**Figure 5:**
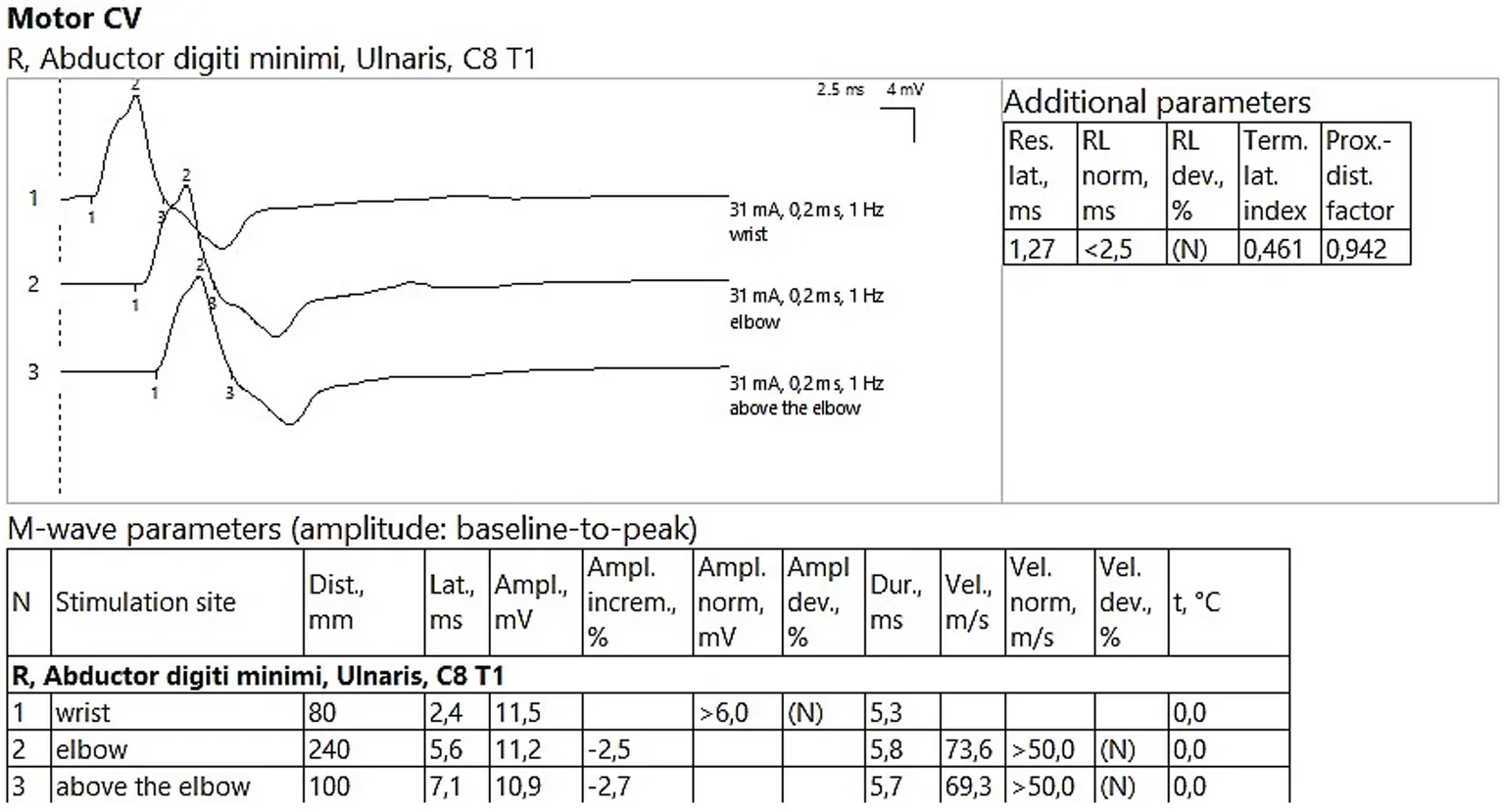
Ulnaris → ADM motor response (Case 2). Ulnar nerve stimulation at the wrist, 2 cm distal to the medial epicondyle, and 10 cm proximal to the medial epicondyle elicited a normal CMAP recorded over the ADM. Distal motor latency was 2.4 ms, amplitude 11.5 mV, and wrist–elbow conduction velocity 73.6 m/s, confirming preserved ulnar motor function in moderate CTS.

**Figure 6:**
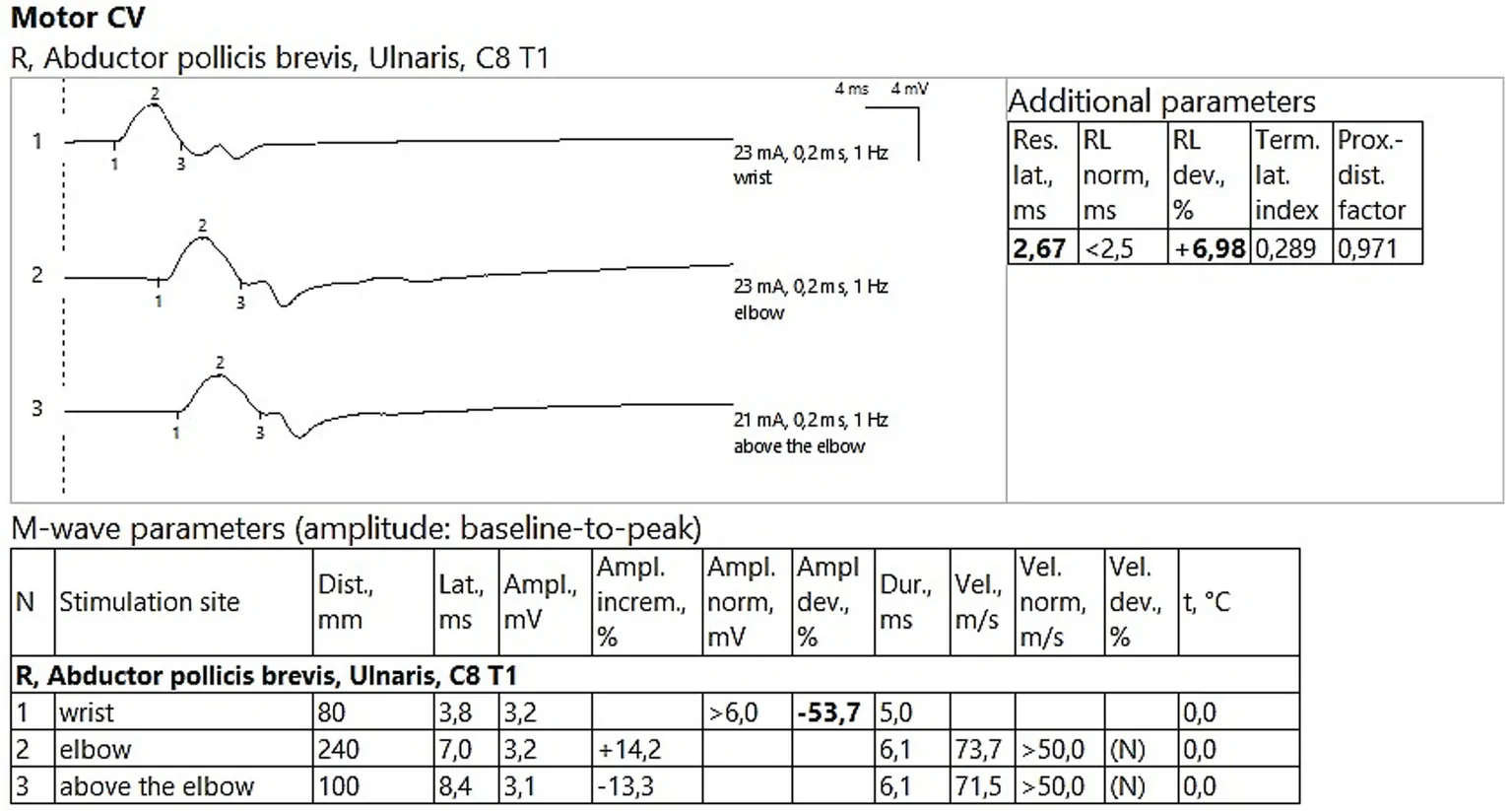
Ulnaris → APB region motor response (Case 2) ulnar nerve stimulation at the wrist, 2 cm distal to the medial epicondyle, and 10 cm proximal to the medial epicondyle elicited a reproducible CMAP recorded over the APB region. Distal motor latency was 3.8 ms, amplitude 3.2 mV, and wrist–elbow conduction velocity 73.7 m/s, indicating prominent ulnar-evoked APB-region activity in moderate CTS.

Waveform documentation (Figures 4–6). Medianus → APB (Figure 4): reduced amplitude and mildly prolonged latency, compatible with moderate CTS. Ulnaris → ADM (Figure 5): normal ulnar motor conduction parameters. Ulnaris → APB (Figure 6): prominent, sharply contoured, reproducible ulnar-evoked APB-region activity.

#### 4.1.3 Case 3—severe CTS with strong ulnar-evoked APB-region activity

A 61-year-old man presented with severe carpal tunnel syndrome, mild thenar atrophy, and reduced thumb abduction strength. Electrophysiological testing demonstrated advanced median nerve involvement with absent median sensory responses and marked impairment of median motor conduction. Despite the severity of the median neuropathy, routine ulnar sensory and motor conduction studies remained preserved.

Targeted ulnar stimulation while recording over the APB region elicited a strong, sharply contoured, and highly reproducible response. The response persisted during proximal stimulation and demonstrated latency characteristics comparable to those observed in the internal physiological comparator dataset. Notably, robust ulnar-evoked activity was detectable at the APB recording site despite marked reduction of the median-evoked APB response. This finding illustrates that pronounced ulnar-evoked APB-region activity may be present even in severe CTS.

**Figure 7:**
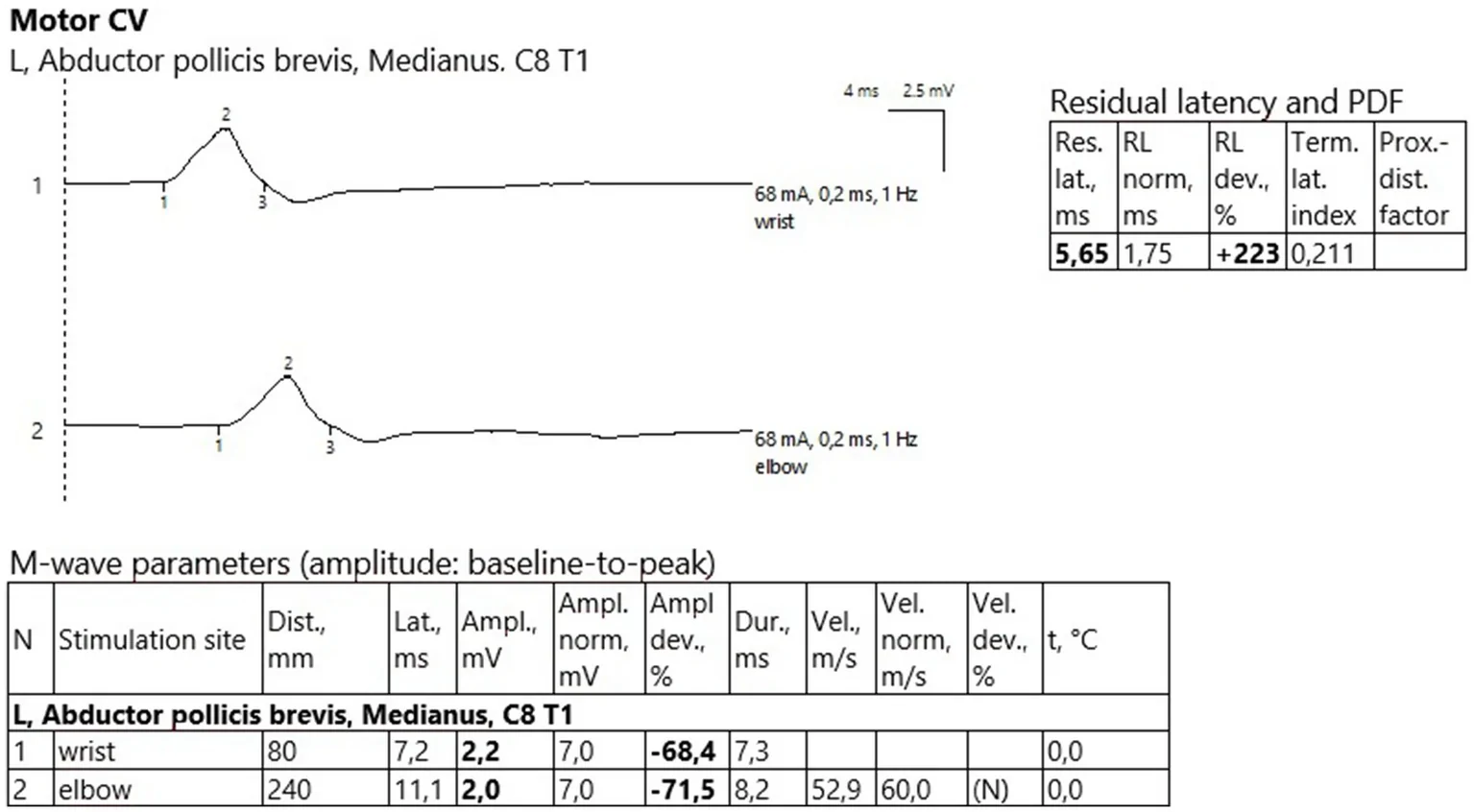
Medianus → APB motor response (Case 3). Median nerve stimulation at the wrist and elbow elicited a markedly impaired CMAP recorded over the APB. Distal motor latency was 7.2 ms, amplitude 2.2 mV, and wrist–elbow conduction velocity 52.9 m/s, consistent with severe CTS and advanced median motor involvement.

**Figure 8:**
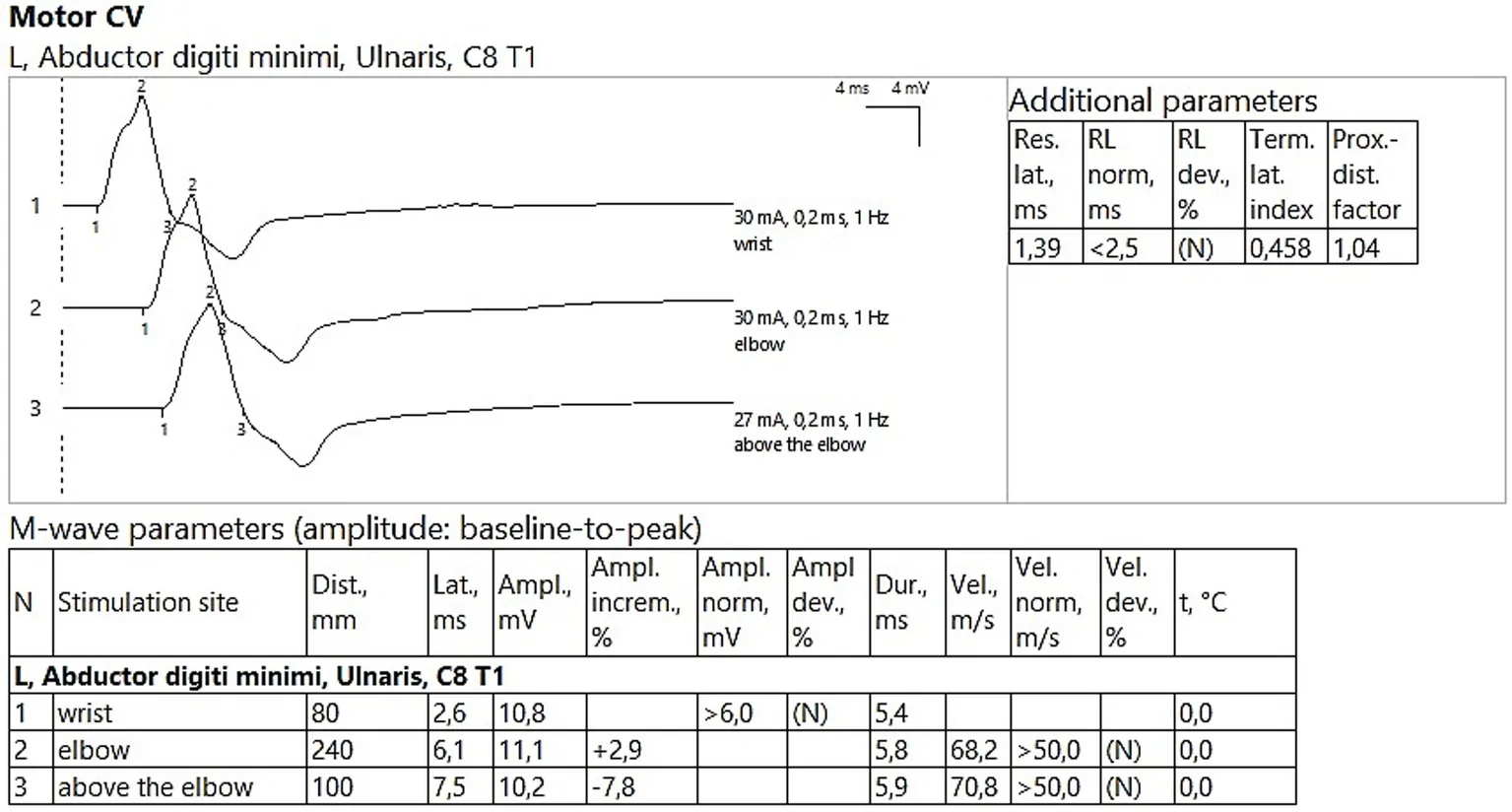
Ulnaris → ADM motor response (Case 3). Ulnar nerve stimulation at the wrist, 2 cm distal to the medial epicondyle, and 10 cm proximal to the medial epicondyle elicited a normal CMAP recorded over the ADM. Distal motor latency was 2.6 ms, amplitude 10.8 mV, and wrist–elbow conduction velocity 68.2 m/s, confirming preserved ulnar motor function in severe CTS.

**Figure 9:**
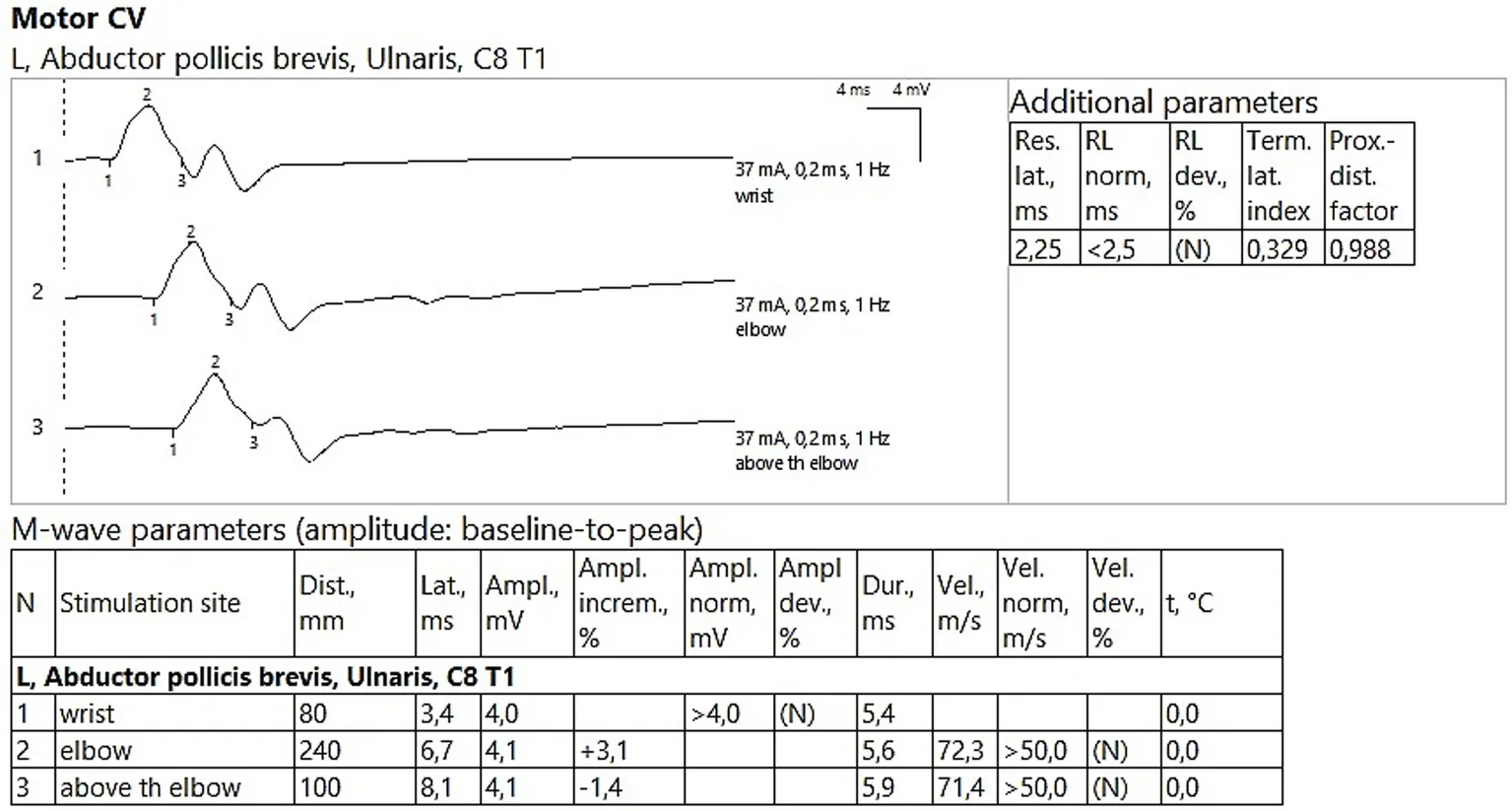
Ulnaris → APB region motor response (Case 3). Ulnar nerve stimulation at the wrist, 2 cm distal to the medial epicondyle, and 10 cm proximal to the medial epicondyle elicited a reproducible CMAP recorded over the APB region. Distal motor latency was 3.4 ms, amplitude 4.0 mV, and wrist–elbow conduction velocity 72.3 m/s, indicating a strong ulnar-evoked APB-region signal in severe CTS.

Waveform documentation (Figures 7–9). Medianus → APB (Figure 7): markedly reduced amplitude and prolonged latency, consistent with severe CTS. Ulnaris → ADM (Figure 8): normal ulnar motor conduction parameters. Ulnaris → APB (Figure 9): strong, sharply contoured, reproducible ulnar-evoked APB-region activity.

### 4.2 Evidence for ulnar-evoked APB-region activity

Across the three CTS-positive cases, reproducible ulnar-evoked activity was recorded over the APB region despite substantial differences in median motor conduction parameters. In mild CTS (Case 1), a small but reproducible response was observed. In moderate CTS (Case 2), a larger and well-formed response was detected, while in severe CTS (Case 3), marked median motor impairment coexisted with a strong ulnar-evoked response recorded at the APB site.

Because the cases were intentionally selected to illustrate distinct electrophysiological presentations, no inference can be made regarding a quantitative relationship between CTS severity and the magnitude of ulnar-derived activity. Rather, the findings demonstrate that ulnar-evoked activity detectable over the APB region may be observed across different CTS severity grades.

The responses demonstrated stable morphology, reproducibility, and persistence with proximal stimulation. These features support the presence of a physiological source of ulnar-evoked activity measurable at the APB recording site. However, contributions from adjacent ulnar-innervated muscles cannot be fully excluded.

Importantly, no sensory response was obtained from digit II during ulnar stimulation in any case. This finding supports the interpretation that the recorded responses were motor rather than sensory in origin, although it does not by itself establish the specific muscle source of the recorded activity.

### 4.3 Proximodistal consistency

The reproducibility of ulnar-evoked activity recorded over the APB region across all three illustrative cases suggests the presence of a physiological motor phenomenon worthy of further investigation. Several anatomical and electrophysiological observations support this interpretation.

First, the intrinsic hand muscles exhibit substantial anatomical variability, and multiple studies have documented median–ulnar motor communications within the hand. Such anatomical variants may provide a plausible substrate for ulnar-derived activity detectable at the APB recording site.

Second, the observed responses demonstrated stable waveform morphology and remained detectable during proximal stimulation. These findings are compatible with activation of a physiologically meaningful motor pathway. Nevertheless, surface recordings cannot determine the exact muscle of origin, and contributions from nearby ulnar-innervated muscles—including the adductor pollicis, the deep head of the flexor pollicis brevis, and the first dorsal interosseous—remain possible.

Third, no sensory responses were obtained from digit II during ulnar stimulation. This observation supports a motor rather than sensory origin for the recorded potentials, although it does not resolve the question of the precise anatomical generator.

Taken together, these findings support the presence of reproducible ulnar-evoked motor activity measurable over the APB recording region. However, additional methods such as needle electromyography or high-resolution ultrasound would be required to determine the exact anatomical substrate of the recorded responses.

### 4.4 Key observation

The three illustrative cases demonstrated reproducible ulnar-evoked activity detectable over the APB region despite substantial differences in median motor conduction parameters. Because these cases were intentionally selected to represent distinct electrophysiological presentations rather than to form a severity-stratified cohort, no inference can be made regarding a quantitative relationship between CTS severity and the magnitude of ulnar-derived activity. Instead, the observations highlight that detectable ulnar-evoked APB-region activity may coexist with varying degrees of median motor involvement across the CTS spectrum.

Across all three cases, ulnar stimulation produced well-formed, stable, and reproducible responses recorded over the APB region. This consistency suggests that the pattern of motor activity detectable at the APB recording site cannot be fully characterized by median motor conduction measures alone. Rather, the findings indicate that under certain anatomical or physiological conditions, ulnar-evoked motor activation may be detectable at the APB site independently of the degree of median dysfunction.

These observations are descriptive and hypothesis-generating. They should not be interpreted as evidence of functional compensation, adaptive reinnervation, or preserved thenar function. Instead, they provide initial electrophysiological support for the existence of ulnar-evoked APB-region activity, warranting further systematic investigation in larger cohorts.

## 5 Discussion

This preliminary case series demonstrates that targeted ulnar motor stimulation can elicit reproducible motor responses recorded over the abductor pollicis brevis (APB) region in selected patients with carpal tunnel syndrome (CTS). Across all three illustrative cases, ulnar stimulation generated stable compound muscle action potentials recorded from the APB site despite substantial differences in median sensory and motor conduction findings. These observations suggest that motor activity measurable at the APB region may not always be fully characterized by median nerve conduction parameters alone.

The principal observation of this study is the reproducible detection of ulnar-evoked motor activity over the APB recording region in patients representing different electrophysiological stages of CTS. In the mild case, the response was small but clearly reproducible; in the moderate and severe cases, larger responses were observed despite progressive impairment of median nerve function. Because the cases were intentionally selected to illustrate different electrophysiological presentations, these findings should not be interpreted as evidence of a relationship between CTS severity and the magnitude of ulnar-derived activity. Rather, they demonstrate that ulnar-evoked activity detectable at the APB recording site may be encountered across the CTS spectrum.

Several anatomical mechanisms could account for the observed recordings. Median–ulnar motor communications within the hand are well documented, and variants compatible with the Riche–Cannieu anastomosis have been described in both anatomical and electrophysiological studies. Such connections provide a plausible explanation for ulnar-derived activation detectable in the thenar region. However, the present study was not designed to establish the precise anatomical substrate of the recorded responses, and the findings should not be interpreted as proof of a Riche–Cannieu anastomosis in any individual patient.

Several electrophysiological features argue against simple distant volume conduction as the sole explanation for the observed responses. The recorded potentials demonstrated physiological distal latencies, reproducible morphology, persistence with proximal stimulation, and consistent proximodistal latency progression. Furthermore, no sensory response was obtained from digit II during ulnar stimulation in any case, supporting a motor rather than sensory origin of the recorded potentials. Nevertheless, surface recordings cannot determine the exact muscle generating the response. Activity originating from nearby ulnar-innervated muscles—including the adductor pollicis, the deep head of the flexor pollicis brevis, the first dorsal interosseous, or other intrinsic hand muscles—may contribute to the recorded signals. Accordingly, the present findings should be interpreted as evidence of ulnar-evoked motor activity detectable over the APB region rather than confirmation of APB-specific innervation.

An additional strength of the present report is the inclusion of a CTS-negative physiological comparator dataset obtained using the same stimulation and recording configuration. The comparator cohort demonstrated measurable ulnar-evoked responses recorded over the APB region with characteristic latency and amplitude ranges, providing a useful physiological context for interpretation of the illustrated CTS cases. Because the comparator dataset was retrospective and was not designed as a normative reference population, these values were used descriptively rather than as diagnostic thresholds.

The targeted maneuver used in this study is simple, rapid, and easily incorporated into routine ENG protocols. In cases where median-evoked APB responses are markedly reduced or absent, the maneuver may provide supplementary neurophysiological information by demonstrating additional ulnar-evoked motor activity at the same recording site. Such information may assist in the interpretation of electrophysiological findings but should not be considered evidence of preserved thenar function or functional compensation.

Several limitations should be acknowledged. The study is based on only three illustrative cases and was not designed to estimate prevalence or diagnostic accuracy. Case selection was retrospective and intended to demonstrate electrophysiological variability rather than to represent a consecutive severity-stratified cohort. In addition, surface recordings do not permit definitive identification of the activated muscle, and complementary techniques such as needle electromyography or high-resolution ultrasound would be required to determine the precise anatomical origin of the recorded activity.

Overall, these observations support the existence of reproducible ulnar-evoked motor activity detectable over the APB recording region in selected CTS patients. Although the anatomical source and clinical significance of this phenomenon remain uncertain, the findings suggest that targeted ulnar stimulation may provide useful supplementary information during routine electrophysiological evaluation. Future studies involving larger cohorts and complementary anatomical or electrophysiological techniques will be required to determine the prevalence, anatomical basis, and potential clinical relevance of this observation.

## 6 Limitations

This preliminary case series has several limitations. The sample size is small, and the illustrative CTS cases were selected retrospectively to demonstrate distinct electrophysiological patterns rather than to represent a severity-stratified cohort. As a result, no conclusions can be drawn regarding the prevalence or severity-dependent characteristics of ulnar-evoked APB-region activity.

Although the targeted ulnar stimulation maneuver was performed in both CTS-positive and CTS-negative limbs, detailed analysis focused on the CTS-positive cases. In the CTS-negative comparator cohort, reproducible ulnar-evoked APB-region responses were present in 13 of 28 limbs, providing physiological context but not constituting a normative reference population.

Surface recordings cannot determine the exact muscle of origin. While the electrophysiological features argue against simple distant volume conduction, contributions from nearby ulnar-innervated muscles—including the adductor pollicis, the deep head of the flexor pollicis brevis, and the first dorsal interosseous—cannot be excluded. Needle EMG or high-resolution ultrasound would be required to confirm the anatomical generator.

Future studies should evaluate the reproducibility of this maneuver in larger cohorts, assess its prevalence across CTS severity grades, and integrate imaging or needle EMG to better characterize the underlying motor pathways.

## 7 Conclusion

This preliminary case series demonstrates that targeted ulnar nerve stimulation can reveal reproducible motor activity recorded over the abductor pollicis brevis (APB) region in selected patients with carpal tunnel syndrome (CTS). Ulnar-evoked responses were observed across mild, moderate, and severe CTS presentations, including cases with markedly reduced median-evoked APB responses.

The findings suggest that electrophysiological activity detectable at the APB recording site may, in some individuals, reflect contributions beyond routine median motor activation alone. Although the precise anatomical substrate cannot be determined from surface recordings, the observed responses were reproducible, physiologically plausible, and demonstrated consistent proximodistal characteristics.

The described maneuver is simple, rapid, and readily incorporated into routine ENG protocols. In selected CTS cases—particularly when median-evoked APB responses are markedly reduced or absent—it may provide complementary neurophysiological information and help identify patterns of ulnar-derived motor activity detectable over the thenar recording region.

These observations should be regarded as descriptive and hypothesis-generating. Further studies combining larger cohorts with complementary techniques such as needle electromyography and high-resolution ultrasound will be necessary to determine the prevalence, anatomical basis, and potential clinical significance of this phenomenon.

## Data Availability

The original contributions presented in the study are included in the article/supplementary material, further inquiries can be directed to the corresponding author.
